# Impact of reactivity controlled compression ignition (RCCI) mode engine operation in diesel engine powered with B20 blend of waste cooking oil biodiesel

**DOI:** 10.1038/s41598-023-31044-6

**Published:** 2023-03-23

**Authors:** M. Anish, J. Jayaprabakar, P. Bency, Nivin Joy, V. Jayaprakash, K. Arunkumar, J. Aravind Kumar, T. R. Praveenkumar, Ayman A. Ghfar, M. Rajasimman, Balasubramani Ravindran

**Affiliations:** 1 School of Mechanical Engineering, Sathyabama Institute of Science and Technology, Jeppiar Nagar, Chennai, India; 2 Department of Electrical and Electronics Engineering, SRM Valliammai Engineering College, Chennai, India; 3 Department of Mechanical Engineering, Assistant Professor, CMR Institute of Technology, Bengaluru, India; 4 Department of Energy and Environmental Engineering, Saveetha School of Engineering, SIMATS, Chennai, 602105 India; 5 Department of Construction Technology and Management, Wollega University, Nekemte, Ethiopia; 6 Department of Chemistry, College of Science, King Saud University, P.O. Box 2455, Riyadh, 11451 Saudi Arabia; 7 Department of Chemical Engineering, Annamalai University, Annamalai Nagar, Chidambaram, Tamilnadu India; 8 Department of Environmental Energy and Engineering, Kyonggi University, Yeongtong-gu, Suwon, Gyeonggi-do 16227 Republic of Korea

**Keywords:** Mechanical engineering, Environmental impact

## Abstract

The purpose of this study is to conduct an experimental assessment of the impact of RCCI (reactivity regulated compression ignition) on the performance, emissions, and combustion of a CRDI engine. A fuel mix (20% biodiesel, 80% diesel, and a NaOH catalyst) is generated. The produced combination is evaluated for attributes using standards established by the American Society for Testing and Materials (ASTM). The engine research included three distinct kinds of injections: 10% Pen RCCI, 20% Pen RCCI, and 30% Pen RCCI. Increasing the injection pressure increases the brake thermal efficiency, often known as BTE. NOx emissions increased as a consequence of higher injection pressures and improved combustion. However, when the injection rate is increased, the Specific Fuel Consumption (SFC) falls. The CO_2_ and hydrocarbon emissions, as well as the smoke opacity values, increased as the charge increased. The resultant mixture may be utilized in a CI engine with pre-mixed ignition to improve overall engine performance as well as combustion characteristics.

## Introduction

The transportation industry accounts for a significant portion of worldwide energy consumption and greenhouse gas emissions^1^. As a consequence, attaining high energy efficiency via rigorous compliance with emission requirements should be at the center of any plan to assure steady, environmentally responsible, and socially fair economic growth^2^. The demand for raw petroleum is expanding at a fast speed as a result of worldwide economic expansion. The haphazard use of different fuel sources has resulted in a rise in the number of people suffering from respiratory problems, as well as an increase in the depletion of fossil fuels, and so on. These factors have paved the path for the use of alternative energy sources[3,4,5,].According to the numerical simulations, increasing the working temperature of the electrochemical cell decreases unit voltage variation and reduces system power usage by 19%^6^. Diesel engines are appealing to researchers because to their high CR and excellent fuel efficiency^7,8^.Regardless, diesel engines have been shown to generate more particulate matter and nitrogen oxides^9^. Because of its reduced emissions and improved efficiency, the premixed charge compression ignition, or PCCI, has prompted concerns in recent years. By using a more advanced mixing of fuel and air before to ignition, particulate matter may be reduced when using the PCCI mode of combustion^10^. NOx emissions were reduced due to the use of a leaner fuel and air mixture in conjunction with an improved exhaust gas recirculation rate (EGR). As a result, the combustion temperature was reduced. Because diesel fuel is more flammable but less volatile than gasoline, there are a few hurdles to overcome in the PCCI mode of combustion. These challenges include producing a homogenous mixture, managing the ignition, having limited functionality, having severe impingement on the combustion chamber walls^11^. It is determined that increasing the volume percentage of particles improves heat capacity and fluid viscosity, however the trend of fluctuations in heat capacity is dependent on the conventional fluids^12^.

The RCCI mode of combustion, a viable and clean-burning technique, was recently invented. To address the challenges associated with the PCCI mode of combustion, this system utilizes two unique kinds of fuels with different physical qualities, as well as separate injection. The phrase "reactivity gradient" refers to the other kind of reactivity, which might be global or local^13^. Both the different kinds of fuel and the quantity of fuel injected into the combustion chamber are used to control global reactivity. The reactivity gradient is distinct from the fuel injection approach, which includes the early and late injection of high and low octane fuels, respectively. As an outcome, the RCCI mode of combustion might vary depending on the fuel injection method and injection rate^14,15^. Higher octane gasoline was used in the intake manifold, while higher cetane fuel was used in the combustion chamber. This was done to arrange the reactivity of the fuel into a separate structure, which resulted in stratified combustion^16^. Using polyoxymethylene dimethyl ethers (PODE) as a high reactivity fuel (HRF) in combination with methanol as a low reactivity fuel (LRF) for RCCI combustion, Duraisamy et al.^17^ greatly reduced the duration of the combustion process and the amount of delay time. Pan et al.^18^ discovered that as the proportion of premixed combustion surged, the IMEP for iso-butanol-diesel, gasoline-diesel RCCI combustion increased significantly. The isobutanol and diesel RCCI engines showed a greater IMEP when both fuels were premixed equally than the gasoline-diesel RCCI engine. Yang et al.^19^ observed that the gasoline and methanol injection timing had an influence on the combustion process in their study on the RCCI engine. It was feasible to improve performance by adjusting the previous diesel injection time and the succeeding methane injection timing. Wang et al.^20^ reported that increasing air intake and decreasing EGR improved the thermal efficiency of a gasoline-PODE RCCI engine. Air dilution to keep intake pressure constant enhanced thermal efficiency. Zheng et al.^21^ demonstrated that low and medium loads lowered RCCI heat output (HRR). Increasing the n-butanol ratio of the engine reduced its thermal efficiency. Charitha et al.^22^ found that adding cotton oil methyl ester reduced NOx emissions. HC emissions increased when the quantity of cotton oil methyl ester was low, but decreased when it was higher. Isik et al.^23^ found that an ethanol-fueled RCCI engine with B50 HRF produced higher peak pressure than the control engine. The HRR curves of the ethanol-fed RCCI engine climbed in all directions. Thiyagarajan et al.^24^ shown that n-pentanol increased BTE when compared to methanol. Dual fuel mode consumed less than biodiesel but more than diesel in terms of brake specific energy (BSEC). The delay time increased with EGR rate and pentanol content, according to Radheshyam et al.^25^. Under low loads, 1-Pentanol decreased cylinder pressure while increased it under high loads.

When used on RCCI, gasoline has a disadvantage since there is insufficient energy available from fossil sources. In addition, the gasoline contains polycyclic aromatic hydrocarbon, commonly known as PAH, which is a factor in the generation of soot^26^. Experts are investigating other fuel sources in an attempt to find a solution to the situation. Because of its oxidation and repeatability for RCCI engines, alcohol fuels have gradually emerged into promising low-activity fuels. This has enabled them to steadily evolve into a suitable low-activity fuel. Furthermore, alcohol energies have higher vaporization idle heat and do not include PAH^27^.Because of their tremendous efficiency and low emissions, RCCI (reactivity regulated compression ignition) engines, which employ the combined fuelling techniques of traditional diesel and gasoline engines, have received a lot of attention^14^. These engines employ a combination of standard diesel and gasoline fuelling techniques. These engines employ a hybrid fueling system, which mixes diesel and gasoline fueling methods. The RCCI was first designed with the intention of employing gasoline and diesel as low and high reactivity fuels to produce the necessary amount of response. Some of the researchers have shifted their focus to determining if RCCI motors can utilise alternative and renewable energy sources. The RCCI was initially designed with the idea of employing gasoline and diesel as low and high reactivity fuels, respectively. Some of the researchers focused their efforts on developing methods to employ alternate and renewable energy sources in RCCI motors. For example, in previous studies, low-reactivity fuels such as natural gas, methanol, and ethanol were employed, although high-reactivity fuels such as diesel were still used^28,29^.

Chen et al.^30^ reported in their study that diesel-n-pentanol-methanol mixed fuels had a shorter combustion duration and a longer ignition delay, resulting in higher HRR when compared to diesel. The diesel-n-pentanol-methanol mixed fuels have lower soot emissions than diesel, but they create greater levels of NOx emissions. Huang et al.^31^ observed that adding n-pentanol enhanced HRR, which accelerated combustion and decreased the time it took for combustion to occur. The proportion of fuel utilized by the brakes grew as the EGR rate increased. Tian et al.^32^ observed that mixing n-butanol with gasoline lowers the temperature of the engine's exhaust gas when compared to using gasoline alone. When compared to pure gasoline, blended fuels including n-butanol and gasoline, as well as pure n-butanol, have the potential to enhance both BTE and volumetric efficiency at low and medium engine speeds. Combining n-butanol with gasoline is likely to lower the quantity of carbon monoxide and nitrogen oxides produced by the engine. Reactivity-controlled compression ignition is a dual-fuel partially premixed combustion concept that employs port fuel injection of a low-reactivity fuel (such as gasoline, gas, and alcohol fuels) and direct injection of a high-reactivity fuel (such as diesel and biodiesel) with blending inside the combustion chamber to increase combustion duration and phasing control^33^. Running dual-fuel engines in RCCI mode improves thermal efficiency while lowering nitrous oxide and soot emissions. The supplied hardware limits stable RCCI combustion. Low loads may lower combustion efficiency, but high loads may result in in-cylinder pressure exceeding the engine's design limit^34^. Under high load, rapid RCCI combustion results in high peak in-cylinder pressures and pressure growth rates^35^.

Waste cooking oils, or WCOs, are beneficial byproducts of the food chain that have the potential to be used as environmentally friendly raw materials in the synthesis of chemicals. Waste cooking oils are also known by their other name, waste cooking oil. The astounding number of WCOs located in various regions of the globe has caused severe issues in the areas of environment, economics, and society. It is estimated that more than 15 million tonnes of waste vegetable oils are created globally each year, with the European Union (EU) contributing over 1 million tonnes annually^36,37^. Triglycerides, monoglycerides, and diglycerides make up the vast bulk of WCOs. Free fatty acids are also found, with quantities ranging from 5% by weight to 20% by weight. Triglycerides, which are primarily composed of saturated and unsaturated fatty acids, are used as platform chemicals in the manufacturing of high-value commodities in a wide range of industries. These platform compounds have a wide range of applications^38,39^. After reviewing a variety of various literature researches, a dependable plan of action was developed and implemented in the current study activity. Because of its efficiency in moderate loads, biodiesel generated from waste cooking oil is a preferable alternative fuel for CRDI engines when compared to efficiency in low or high loads. Biodiesel, in addition to being considered an alternative fuel, has a substantial influence on the quantity of pollution that is avoided from being emitted into the atmosphere when it is burnt. Furthermore, it gives a solution to the dilemma of the demand for fossil fuels. This field of research provides a mechanism for CRDI engines to utilise a variety of waste cooking oils. Furthermore, in order to offer Reactivity Controlled Compression Ignition (RCCI),which guarantees that the diesel engine functions as efficiently as feasible.

## Procedures and entrap for experiment

**Figure 1:**
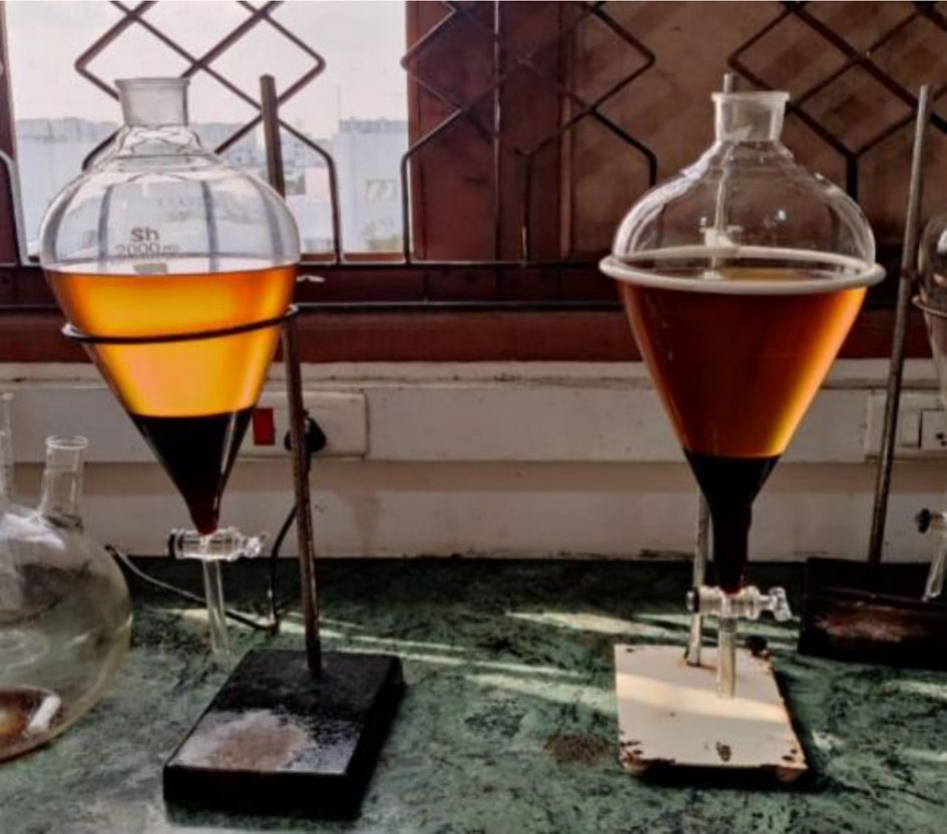
Preparation of bio-diesel.

The combination of 80% diesel (3L) and 20% biodiesel resulted in the creation of a 4L B20 oil mix, which is more frequently referred to as a high performance fuel blend (1L waste cooking oil). During the process of transesterification, we used 1.2 L of oil that had been heated to 60 degrees Celsius. 180 ml methanol and 4.5 g sodium hydroxide catalyst were added. Next that, there is a period of relaxation for the following 90 min. After that, the biodiesel is transferred into the separating funnel so that the layers may be separated, and it is allowed to sit still for two hours while this process is carried out. After that, it will begin to split into two layers, with the glycerine layer located at the bottom and the biodiesel layer located on top. The last process involves washing the biodiesel. In which the temperature of the water is brought up to seventy degrees. Afterward, during the last stage of the processing, the biodiesel is cooked at a constant temperature of 110 degrees for the subsequent 45 min. In order to produce the end product, 1.2 L of used cooking oil are converted into 1 L of bio-diesel which can be shown in Fig. 1.

### Properties validating

The important aim of the property screening is to assess and contrast the fuel blend's physical and chemical properties with those of the standard values. This will be done by comparing the fuel blend to a set of standard values. The fuel combination that has been formed is put through a number of tests utilizing a range of scientific equipment in order to identify its physical and chemical qualities, and the results of these tests are gathered when they have been completed.

#### Density measurement

Hydrometer, It is a piece of machinery that determines the specific gravity of a material. It is precise to 0.001 degrees. The hydrometer is used to calculate the density of the biodiesel mixture. After selecting an acceptable specific gravity hydrometer, 250 ml of the biodiesel mixture are put into the beaker. After being immersed in the beaker, the hydrometer was exposed to the stabilization procedure. The hydrometer displayed the specific gravity measurement received after being immersed.

#### Viscosity measurement

**Figure 2:**
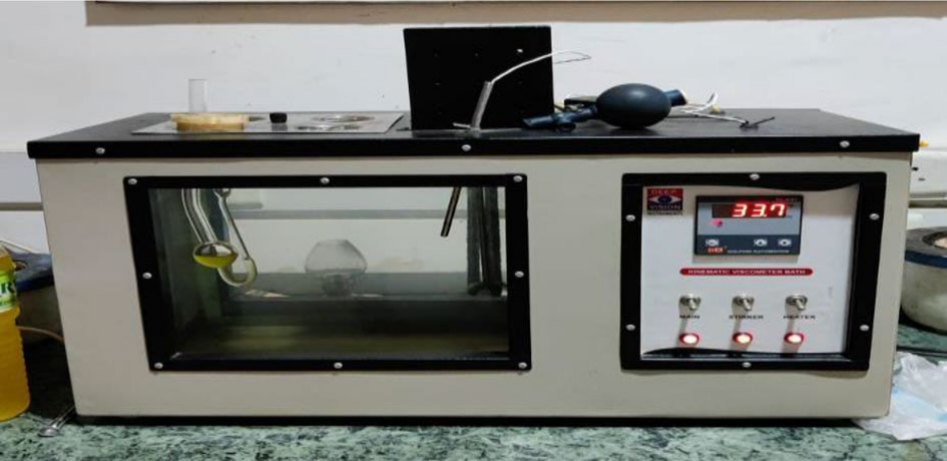
Viscosity measurement.

Viscosity is measured using a Canon-Fenske Viscometer (Fig. 2). The viscosity tube was cleaned with acetone and dried. 50 ml of biodiesel went into the viscosity tube. Suction was needed to raise the gasoline viscometer mark. To warm the biodiesel sample, the viscometer was placed in a 40 °C bath for 30 min. After biodiesel reached the higher point, the suction force was shut off, and the time it took to reach the lower target was recorded.

#### Calorific value measurement

The value of the calorific content is figured out with the use of a Bomb calorimeter. The bomb, fire unit, offset stirrer, gas release valve, water jacket, timer, pressure gauge, crucible, ignition wire, thermometer, and calorimeter vessel are the components that make up the calorimeter. The quantity of heat produced by the combustion of 10 ml of biodiesel mix in an enclosed chamber is used to compute the calorific value of the fuel***.***

## The experiment's set-up and operating stipulations

**Figure 3:**
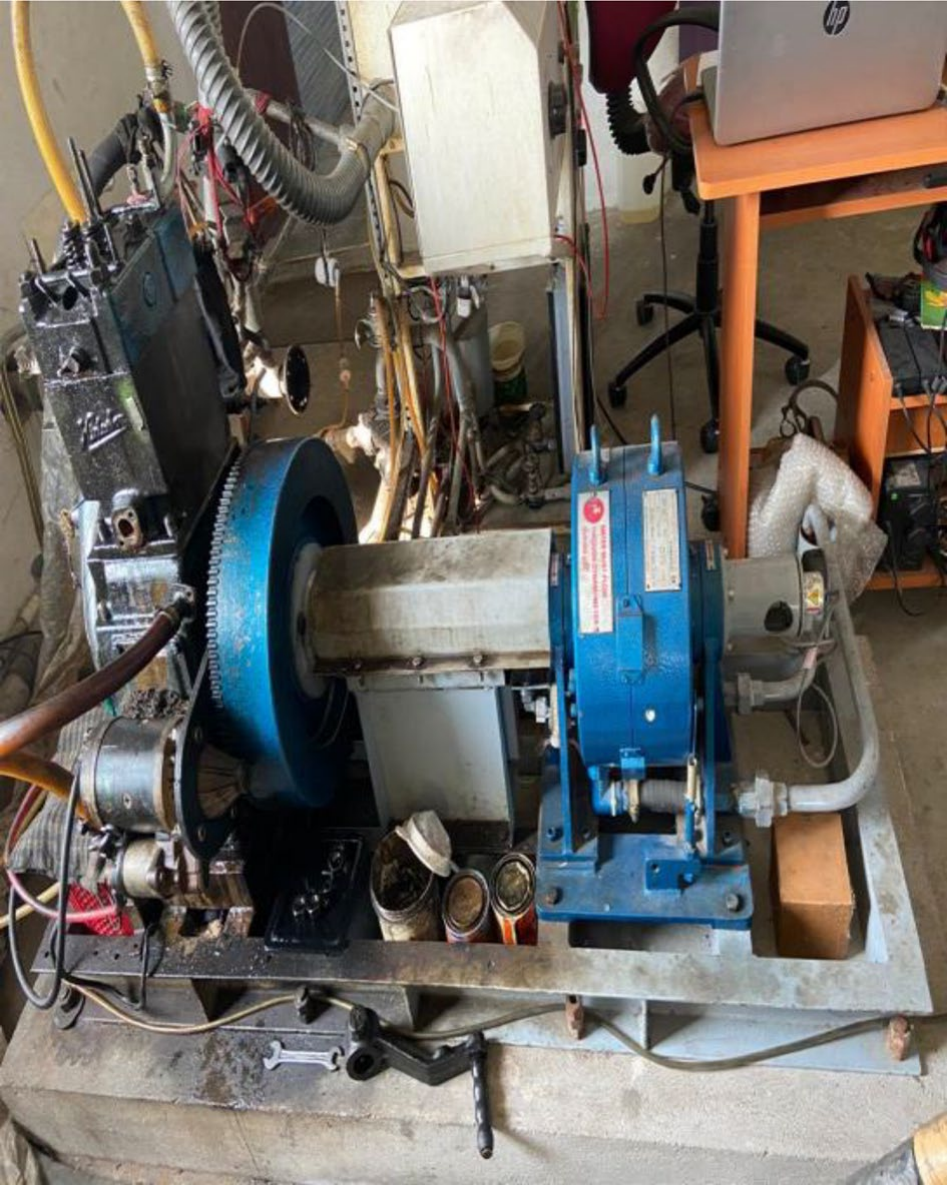
Engine test rig.

A diesel engine with a single cylinder and four strokes is employed for the experimental investigation. This dynamometer employs eddy current to load the engine and is connected to it in order to do so. It comes pre-equipped with the required equipment for detecting things like crank angle and combustion pressure, and it can be utilized right away. A data gathering device comprised of a piezoelectric pressure sensor and a crank angle indicator was developed and installed in the engine to aid in the monitoring of the engine's combustion characteristics. To determine the cylinder pressure, heat release rate, and ignition delay, the data collecting system must average the output signal from a pressure transducer attached to the data acquisition system over a fifty-cycle period. This is required to get an accurate measurement of the cylinder pressure, heat release rate, and ignition delay. Figure 3 depicts the test rig as well as a high-level schematic layout that offers a high-level overview of the whole system. Before any steady state measurements can be obtained, the engine must first be started with no load and then given enough time to warm up to its rated speed of 1,500 revolutions per minute (rpm). When the engine has reached its rated speed of 1500 revolutions per minute, steady state measurements may be obtained. As a result of this research, it will be possible to monitor and analyze the effects of brake thermal efficiency and other emission parameters, such as nitrogen oxides, ethane gas temperature and composition, carbon monoxide concentration and composition, smoke opacity, and smoke vapour pressure, on engine performance and emission levels. It includes a particle size analyzer in addition to a smoky meter. The opacity meters that detect and measure the quantity of light that is obstructed in a sample of smoke given out by diesel engines are known as the diesel exhaust smoke meters. The smoke meter will indicate the smoke density, which will provide an indication of the rate of the combustion's efficiency. A separate electrical control unit is housed in a separate measuring head inside the smoke meter's measuring head. Additionally, the measuring head has an optical unit. The gas analyzers work on the idea that the gas being tested will absorb light in order to provide an accurate reading. The analyzer merely has to shoot a beam of light through the unheated chamber, and then the amount of the particular wavelength that was detected by the sample is measured. The analyzer is made out of an optical filter that blocks out any and all light outside of the wavelength that the particular gas molecule can detect. Other gas molecules do not have the ability to absorb light at this specific wavelength, and they do not even have an impact on the quantity of light that is received by the detector.

After starting the diesel engine with these parameters, the generated test fuels were given to it to operate on so that it could be assessed in accordance with Table 1.The engine was exposed to a range of loads while being tested and collected performance data at a constant speed of 1500 revolutions per minute (rpm).

**Table 1:** Configuration of the engine.

Engine type	RCCI engine
Model	Kirloskar TV1
No. of. cylinders	1
Cubic capacity (liter)	0.661
Rated speed (rpm)	1500
Governing	Class “A2/B1”
Power	5.20 KW
Power rating	7 hp
Cylinder bore	87.50 mm
Stroke length	110.00 mm
Connecting rod length	234 mm
Compression ratio	17.50
Swept volume	661.45 cc
Over all dimensions	Width 2000*length 2500*height 1500 mm

### Accuracy and uncertainties

Uncertainty and inaccuracy may be caused by a variety of factors, such as the selection of testing equipment and how well it is calibrated, as well as the constantly shifting conditions that are present in the environment. The majority of unclear outcomes may be traced back to either premeditated blunders or unanticipated slipups. In an earlier discussion, we discussed the problem of repeatability; in the second discussion, we spoke about the problem of analysis^40^. In the posterior probability of the 2 Guassian distributions, the uncertainty variance in the test statistic, which is denoted by the axis X, is evaluated using the Eq. (1). 95% of the numbers computed fall within a limit of two cents, which is the average.

1 $$\Delta X_{i} = \frac{{2\sigma_{i} }}{{X_{i} }}*100$$

Xi represents the experimental mean and standard deviation. These statements claim that an evaluation of measurement uncertainties has been made^41^.

2 $${\text{R}} = {\text{f}}\left( {{\text{X1}},\;{\text{X2}},\;{\text{X3}} \ldots {\text{Xn}}} \right)$$

3 $$\Delta R = \sqrt {\,\,\left[ {\left( {\frac{\partial R}{{\partial X1}}\Delta X1} \right)^{2} + \left( {\frac{\partial R}{{\partial X2}}\Delta X2} \right)^{2} + \left( {\frac{\partial R}{{\partial X3}}\Delta X3} \right)^{2} + \cdots \cdots \left( {\frac{\partial R}{{\partial Xn}}\Delta Xn} \right)^{2} } \right]}$$

In the equation, the letter 'R' stands in for the function of X1, X2, Xn, and so on (2). The total number of measurements, denoted by Xn, was determined to be. The value of 'R' is determined by taking the square root of the standard deviation that is linked with the defining expectations.

### Ignition Parameters and Their Determination Methods

In order to get a greater understanding of the engine's combustion characteristics, combustion metrics such as IMEP and COV, which demonstrated both thermal efficiency and ignition efficiency, as well as higher in—cylinder temperature, Rate of pressure, and MFB, were employed. According to the conclusions of this study, the IMEP was calculated as follows during both the compression and expansion phases:

4 $$IMEP = \mathop \int \limits_{ - 180CA}^{180CA} PDV\frac{dy}{{dx}}$$

The volume of the displacement, denoted by V_d_, may be calculated by integrating a function beginning at the beginning of the compression stroke (180 degrees CA) and continuing all the way until the conclusion of the expansion stroke (180 deg. CA). The COV was formulated in this manner:

5 $${\text{COV}} = \frac{IMEP\; Standard}{{IMEP \;Average}}$$

## Discussion of the findings

### Brake thermal efficiency

**Figure 4:**
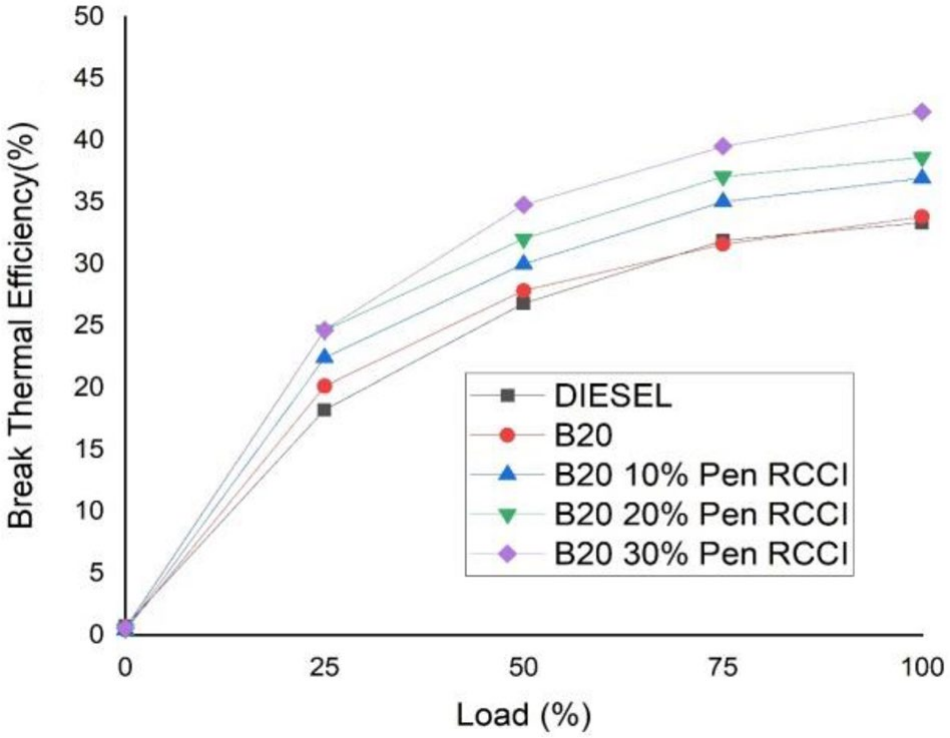
Break thermal efficiency versus load.

The transesterification process is used to improve and assess biodiesel synthesis. The transesterification process lasted around 90 min at 60–65 °C. Excess methanol was employed at a 6:1 methanol-to-oil ratio to increase biodiesel output. KOH was utilized as a catalyst, resulting in a maximum yield of 96%.When several property tests of high performance fuel, Biodiesel, and Diesel are considered, it is discovered that the copper corrosion test yielded the same findings for all kinds of fuels discussed. Cloud point for conventional diesel, biodiesel, and High performance fuel mix is − 1 °C. The calorific value is particularly important throughout the combustion process; greater calorific values are preferable. Figure 4 shows that the calorific values vary relatively little (i.e., diesel-9235.23 Cal/g, biodiesel-7445.65 Cal/g, and high performance fuel blend-8818.116 Cal/g). Similarly, all of the attributes were comparable to those of conventional diesel. High performance gasoline and biodiesel have an extremely high pour point temperature, which means they may be utilized at temperatures as low as − 8 °C. This is a very desired fuel characteristic since many nations, including the United States, Russia, China, and Canada, have temperatures below zero degrees Celsius.

It was observed that there is a large difference in BTE for each machine cargo, up to four points for certain weights. These are the impacts of altering the CRDI engine premixed ratio based on load at a particular BMEP^42^. In all circumstances of mixed fuels, increasing the load increases brake thermal efficiency. The heat loss will decrease as the load rises. According to the graph, B20–30% Pen RCCI has the greatest BTE, followed by B20–20% Pen RCCI, B20–10% Pen RCCI diesel, and B20-normal. The 30% premixed charge obviously has the greatest BTE throughout the injection compared to the B20–20% Pen RCCI, B20–10% Pen RCCI, B20, and Diesel.

### Volumetric efficiency

**Figure 5:**
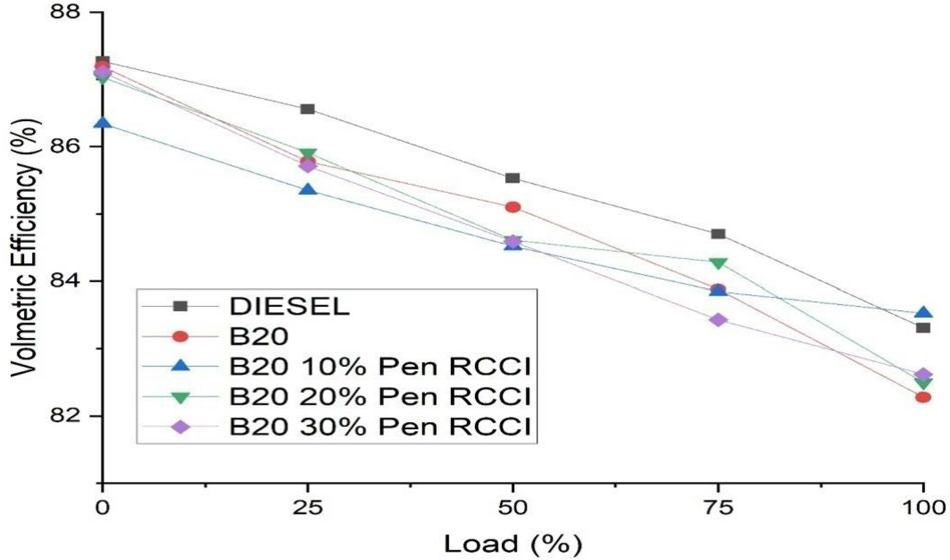
Volumetric efficiency versus load.

The ratio of the quantity of air that is scented by the machine to the amount of air that is swept by the piston is the volumetric performance of the machine. In the expansion stroke, the piston travels to the bottom dead center, but the volume it sucks from the outside is not the same as the volume it swept when expanding. The graph shows that diesel has the maximum volumetric efficiency, however increasing the load shows that volumetric efficiency decreases under different situations. Figure 5 show that B20–10% Pen RCCI has the best volumetric efficiency at maximum load.

### Exhaust gas temperature

**Figure 6:**
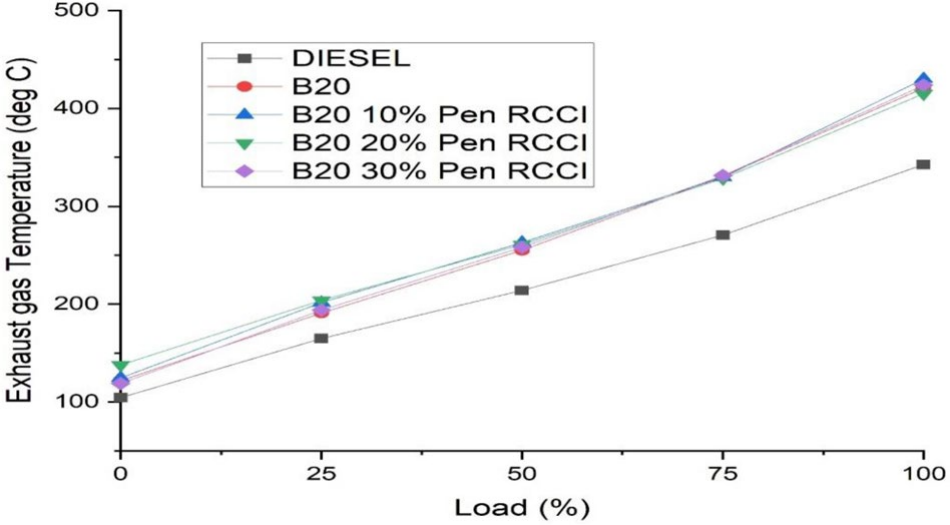
Exhaust gas temperature versus load.

An exhaust gas temperature meter is a device that measures the exhaust gas temperature of an internal combustion engine. Depending on the energy, the temperature of the exhaust gas rises or falls. As demonstrated in Fig. 6, the exhaust gas temperature rises as load increases; at maximum load, RB20 has the greatest exhaust gas temperature compared to other fuels. RB20-PM30% has the lowest exhaust temperature at maximum load.

### Specific fuel consumption

**Figure 7:**
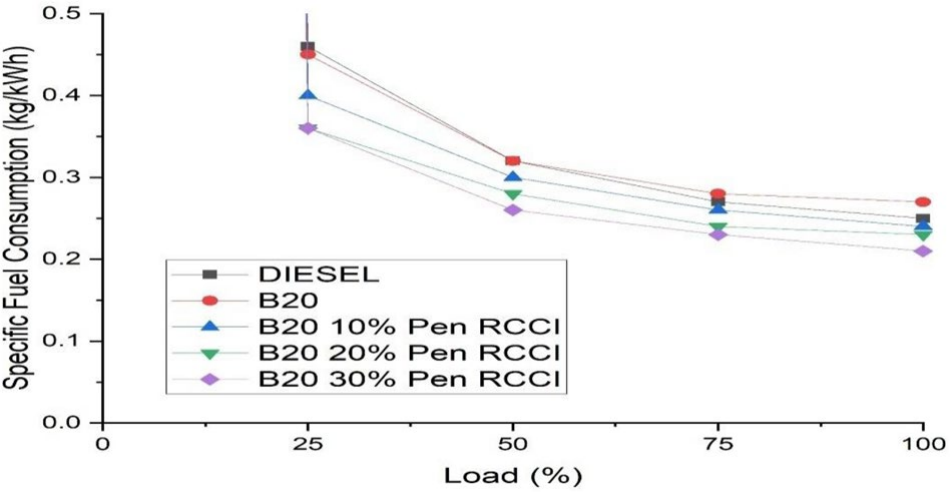
Specific fuel consumption versus load.

It is the quantity of fuel used by a vehicle for each unit of power produced. An engine's specific fuel consumption is the amount of fuel used to create one unit of thrust. It compares the efficiency of CRDI engines. Figure 7 depicts the particular fuel consumption at various loads. As the load rises, so does the specific fuel consumption. When compared to B20–10% Pen RCCI, B20–20% Pen RCCI, diesel, and B20, B20–30% Pen RCCI has the lowest specific fuel usage.

## Emission characteristics

### 1Carbon monoxide

**Figure 8:**
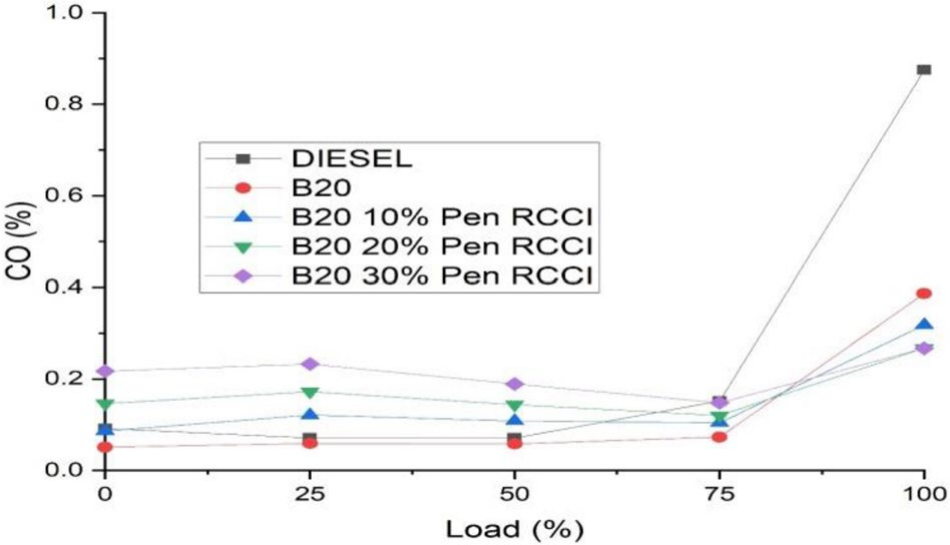
Carbon monoxide versus load.

In an RCCI engine, carbon monoxide is produced as a byproduct of incomplete combustion. It is created by the partial oxidation of carbon-based composites. Following burning, carbon dioxide (CO2) is often produced. Figure 8 demonstrates that diesel has the lowest CO emission, whereas B20 has almost the same. At certain loads, the CO emission is B30–30% Pen RCCI, but beyond that, it is as low as diesel.

### Carbon dioxide

**Figure 9:**
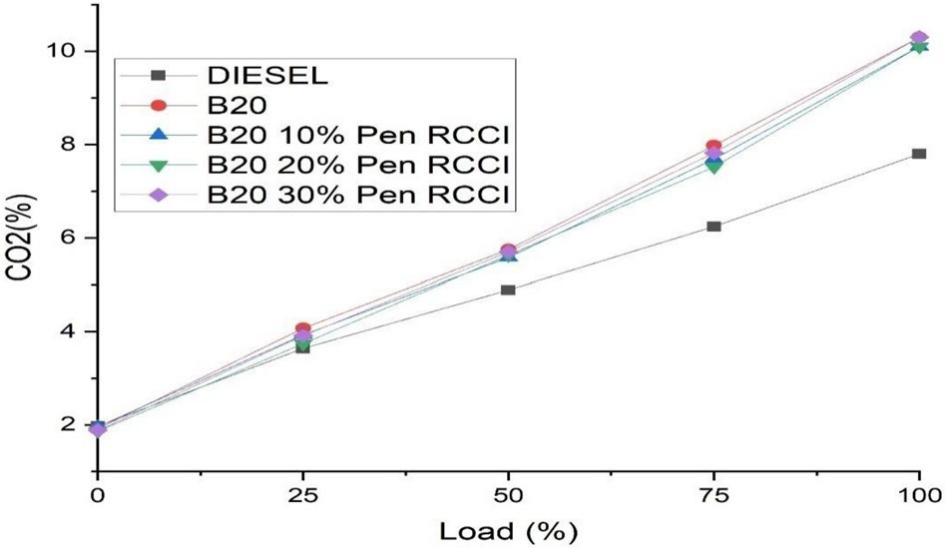
Carbon dioxide versus load.

Carbon and hydrogen atoms are found in gasoline. Carbon dioxide is produced during combustion when carbon (C) from the fuel interacts with oxygen (O2) from the air (CO2). In this Fig. 9, B20–30% Pen RCCI has the lowest emission in the greatest load, and B20 has the highest emission in the lowest load, but most fuels are about the same in the conclusion.

### Nitrogen oxide (NOx)

**Figure 10:**
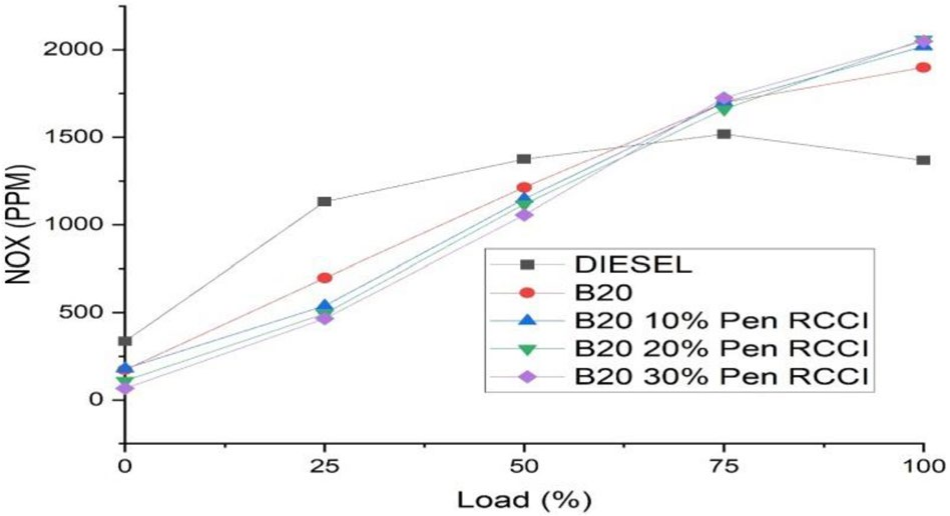
Nitrogen oxide versus load.

CRDI engines run at greater temperatures and pressures than gasoline engines. NOx gases are produced as a result of these situations. Nitrogen oxides are very toxic and highly reactive gases. These gases are produced when high-temperature fuel is burnt. Automobiles, trucks, and other non-road vehicles generate NOx pollution (example construction equipment, boat). It seems to be a brownish gas. In this Fig. 10, diesel has the lowest NOX emissions at both the lowest and greatest loads. When compared to B20, Diesel, B20–20% Pen RCCI, and B20–10% Pen RCCI, B20–30% Pen RCCI has the highest emission.

### Hydrocarbon emission

**Figure 11:**
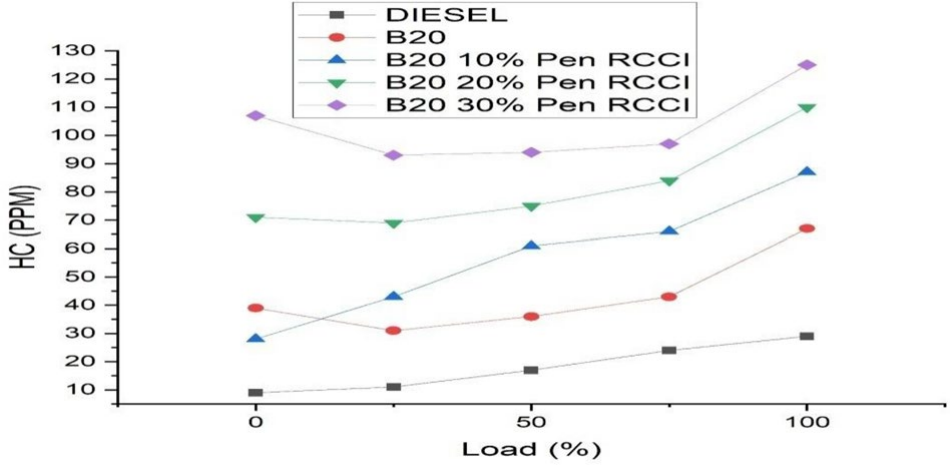
Hydrocarbon emission versus load.

It is mostly caused by incomplete fuel combustion in the combustion chamber. It is just unburned gasoline poured directly into the exhaust system. Compression is reduced when there is an ignition issue or an internal engine breakdown. A substantial amount of HC will be released into the environment as a result of inadequate fuel ignition. Figure 11 depicts the greatest HC emission value at maximum load for the B20–30% Pen RCCI. DIESEL emits the least amount of pollution at both maximum and minimum loads.

### Smoke opacity

**Figure 12:**
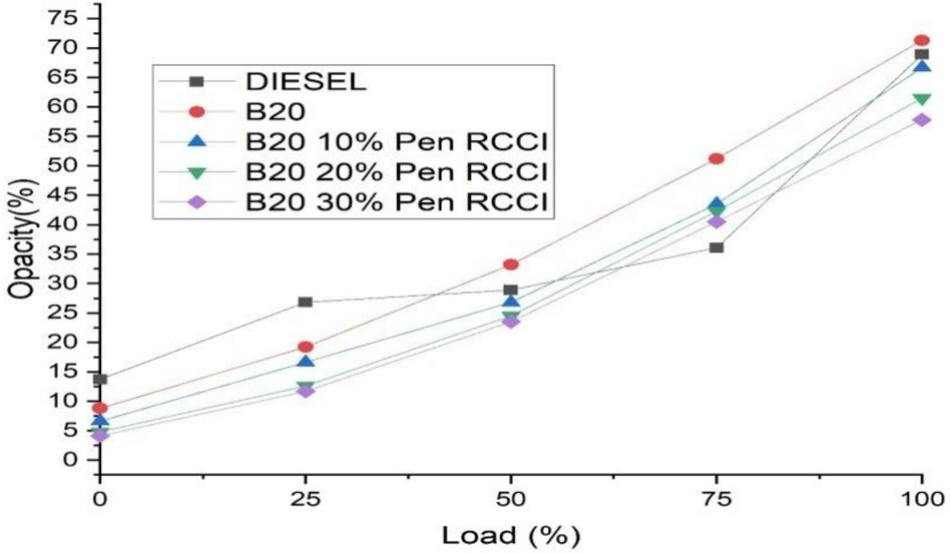
Smoke opacity versus load.

In this form of emission, the particles that are produced are those that are brought about by the deposition of fuel on the cylinder wall. The amount of light that is blocked by smoke is referred to as its opacity, and it plays a role in the measurement of the amount of smoke that is produced by a CRDI engine as a result of gasoline that is left on the cylinder walls. The opacity of B20 is the greatest at both the maximum and the lowest loads, and B20–30%. Figure 12 show that the Pen RCCI has the lowest opacity even when loaded to its full capacity.

## Combustion graphs

### Crank angle versus cylindrical pressure

**Figure 13:**
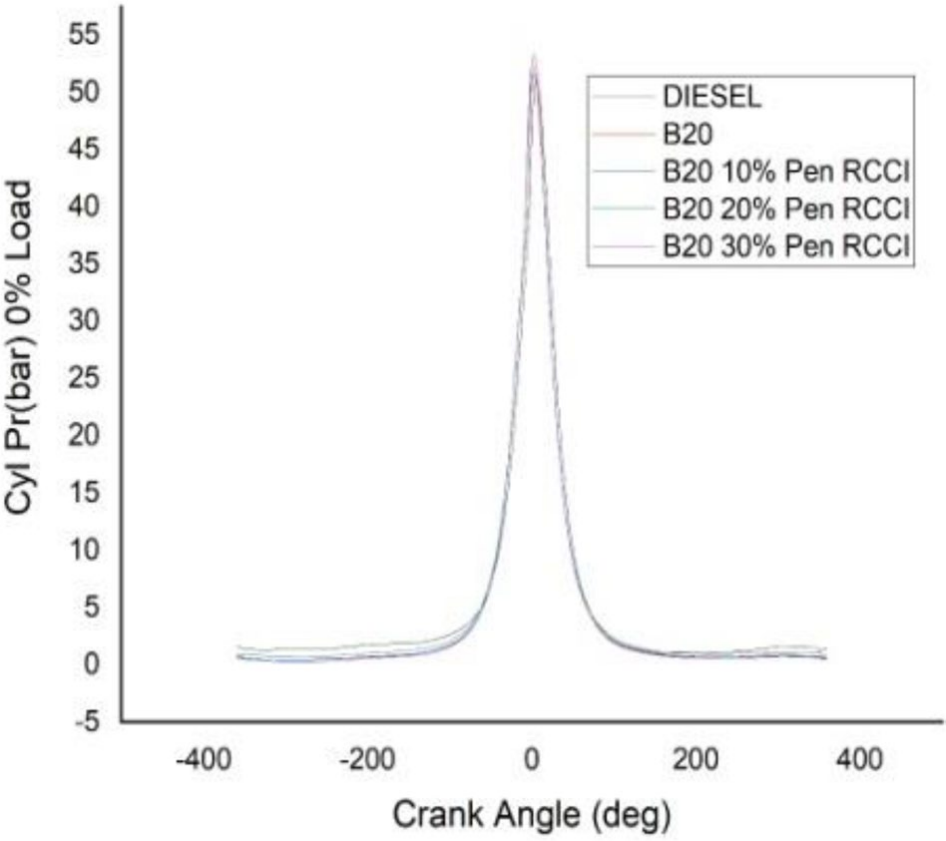
Cylindrical pressure at 0% load.

**Figure 14:**
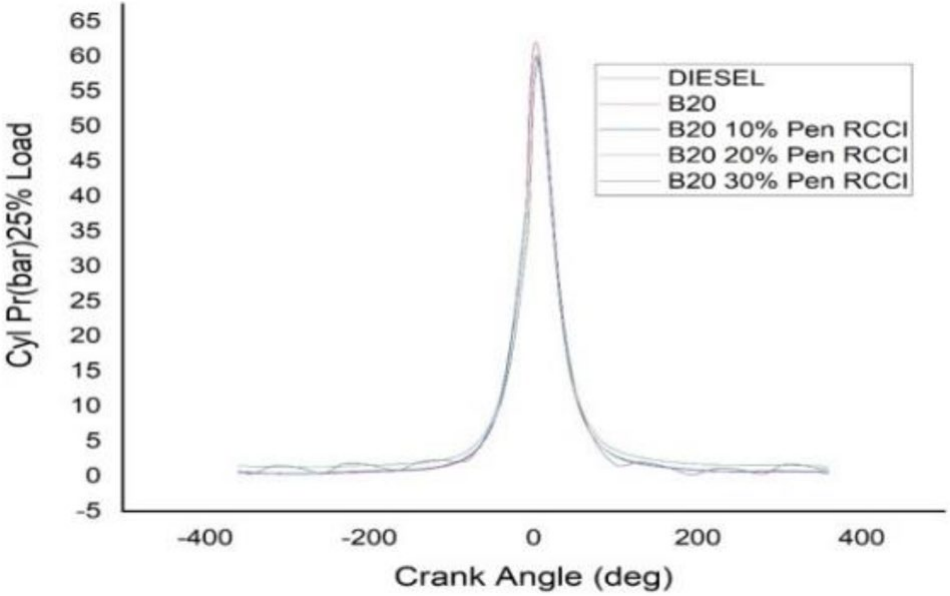
Cylindrical pressure at 25% load.

**Figure 15:**
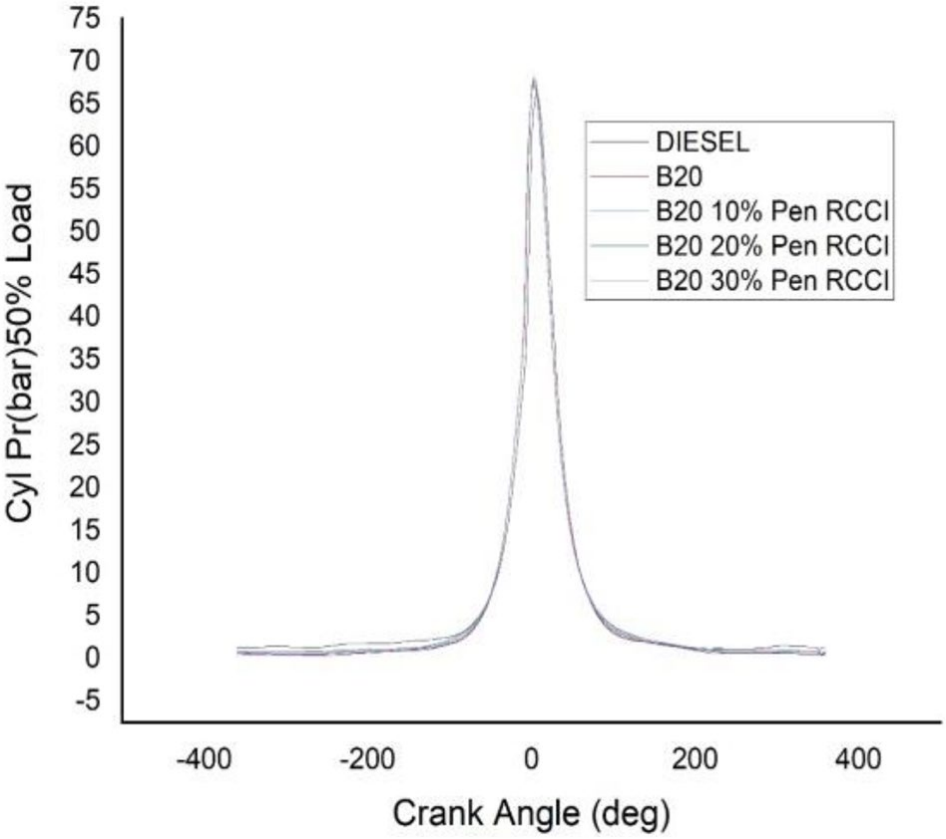
Cylindrical pressure at 50% load.

**Figure 16:**
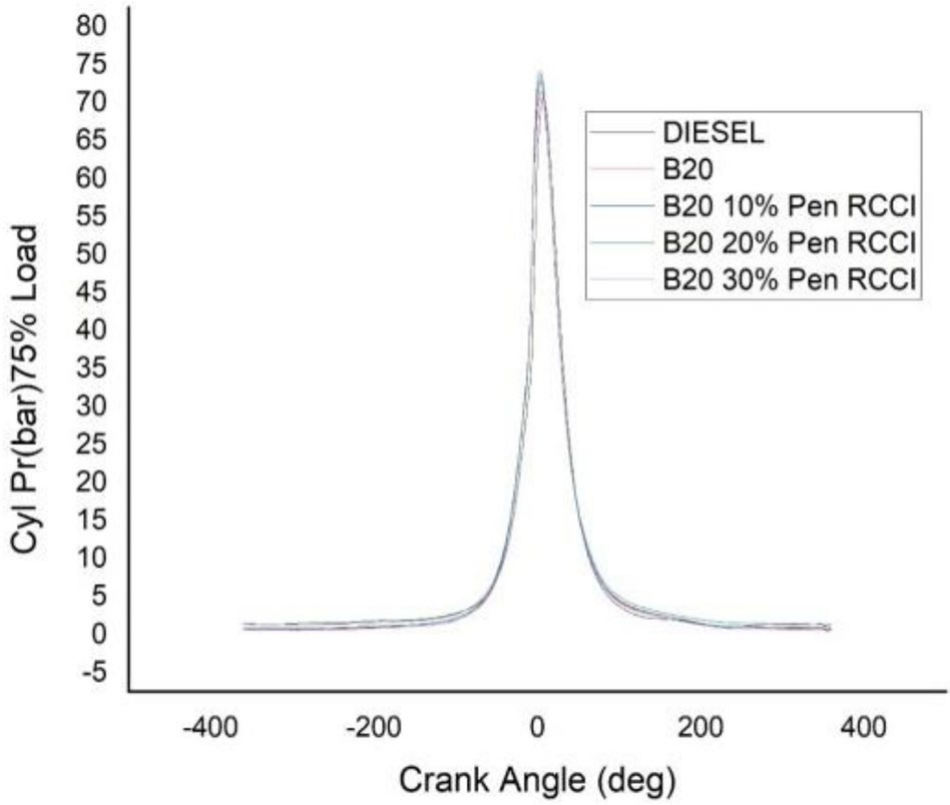
Cylindrical pressure at 75% load.

**Figure 17:**
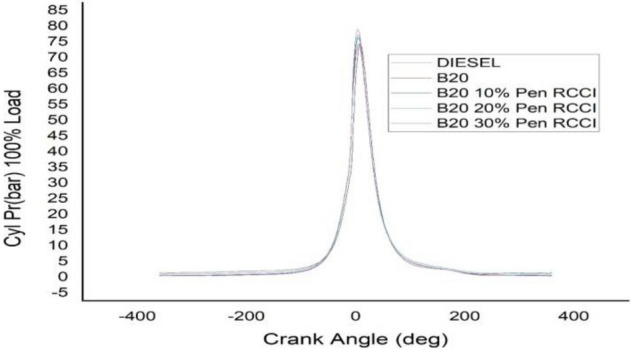
Cylindrical pressure at 100% load.

Figures 13, 14, 15, 16 and 17 depicts crank angle and cylindrical pressure; we can observe that cylindrical pressure is greater in diesel and B20–30% Pen RCCI has the lowest CP at lower loads.When the crank angle increases, the CP for B20–30% Pen RCCI increases. DIESEL has the most CP at 0% load and B20–30% Pen RCCI has the lowest CP. At 25% load, Pen RCCI has the lowest CP and Diesel has the greatest CP. Diesel has the greatest CP at 50% load, followed by B20, and B20–30% Pen RCCI has the lowest CP. At 75% load, B20–30% Pen RCCI has the greatest CP, followed by B20–20% Pen RCCI, and B20 has the lowest. At maximum load (100% load), B20–30% Pen RCCI has the greatest CP while diesel has the lowest CP. As a result, an increase in crank angle is inversely proportional to an increase in cylinder pressure.

### Crank angle versus net HR J/Deg

**Figure 18:**
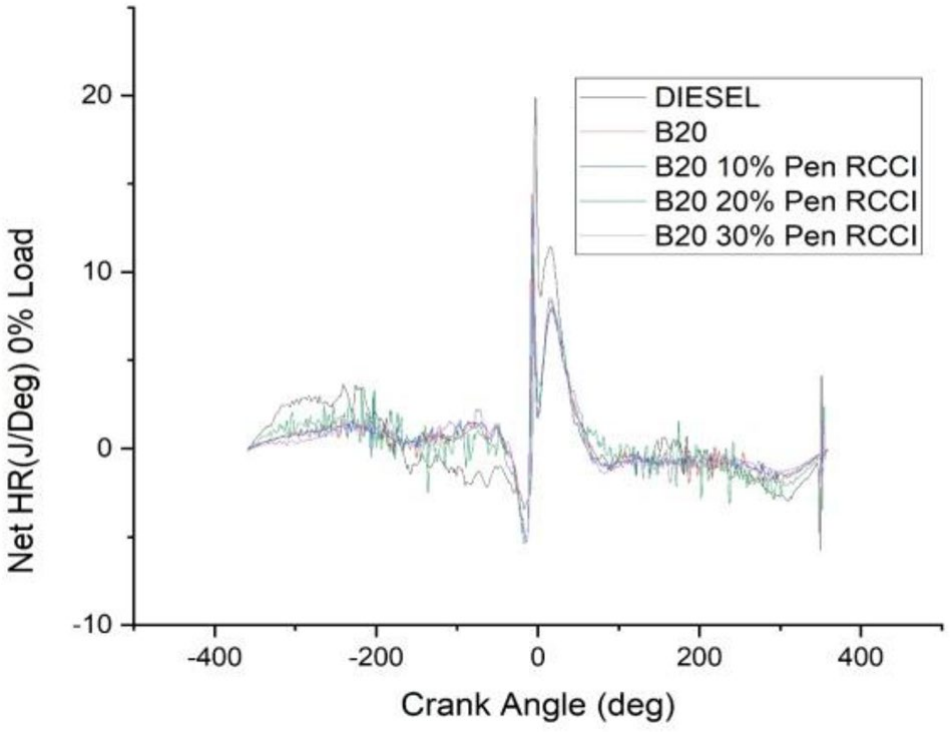
Net heat release at 0% load.

**Figure 19:**
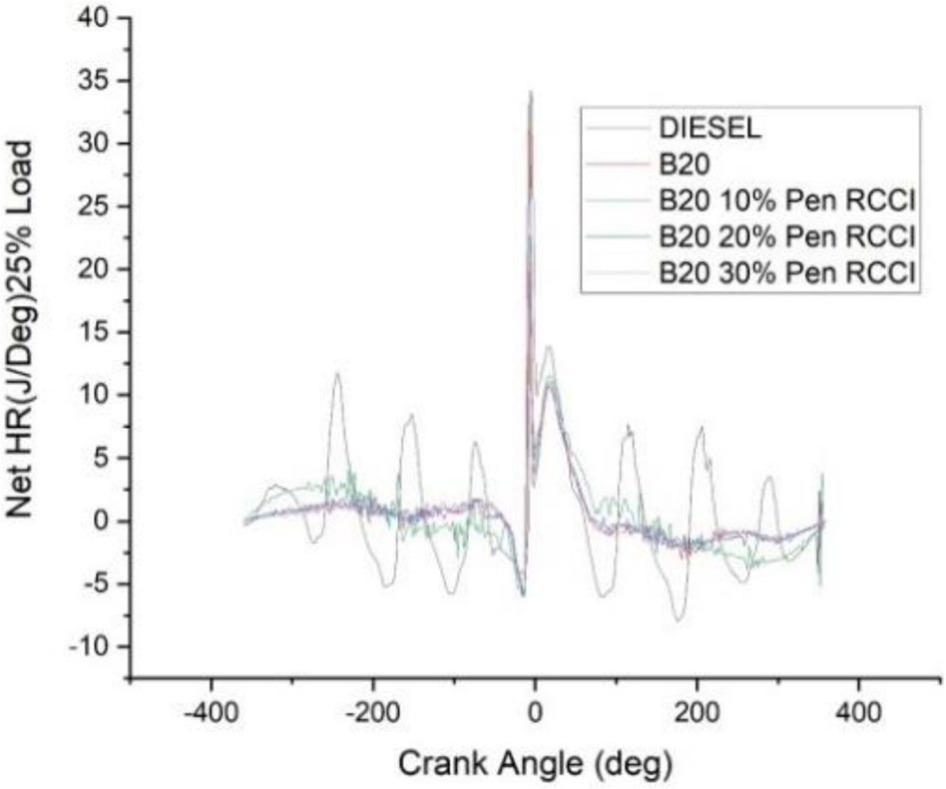
Net heat release at 25% load.

**Figure 20:**
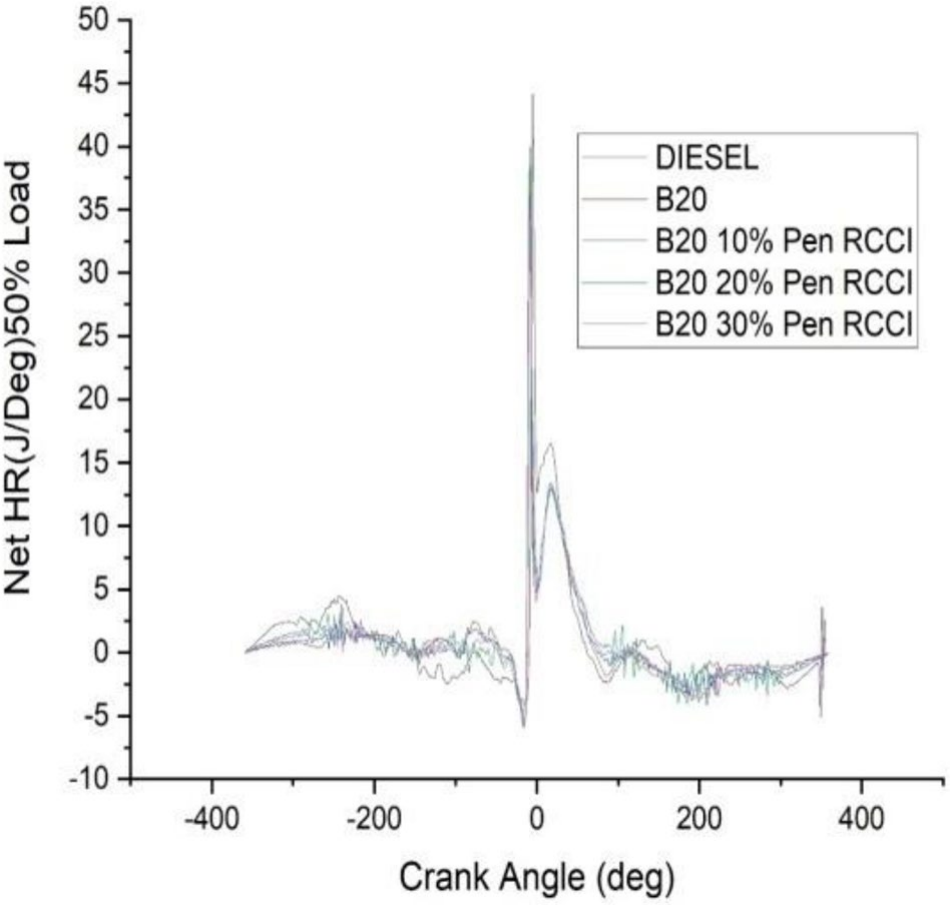
net heat release at 50% load.

**Figure 21:**
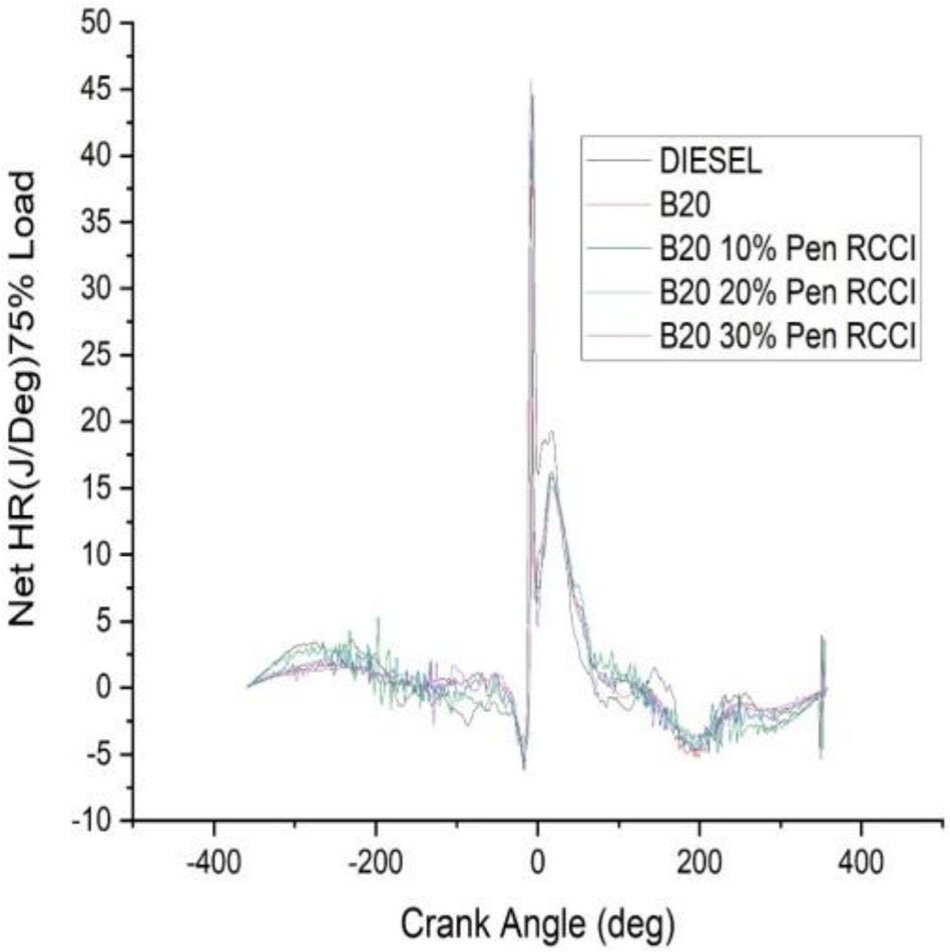
Net heat release at 75% load.

**Figure 22:**
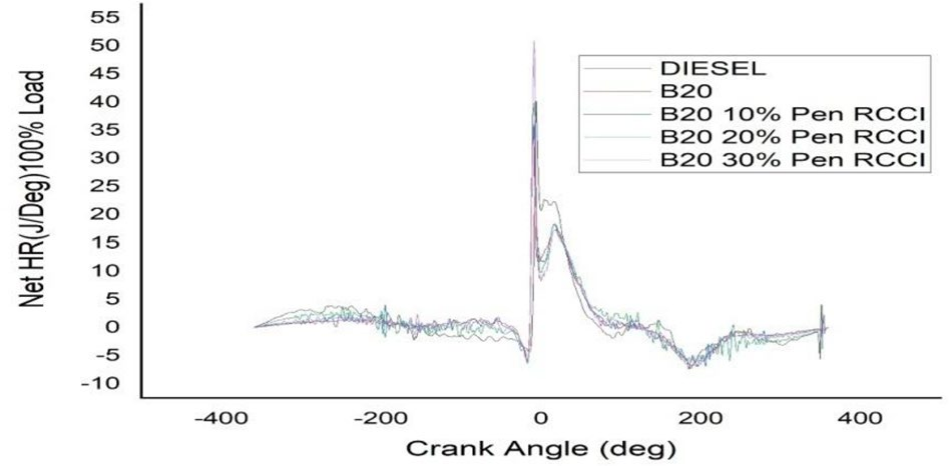
Net heat release at 100% load.

The angle of the crank has an effect on the total amount of heat that is released. According to the data shown in the Figs. 18, 19, 20, 21 and 22 diesel has the highest net heat release at 0% load, followed by B20–10% Pen RCCI, and B20 has the lowest. By increasing the load to 25%, we are able to investigate the oscillations that occur in diesel at various crank angles. B20 is subject to more extreme variations than diesel. The net heat emission from B20 is the highest, while the net heat release from diesel is the lowest. Diesel has the most variability at all loads, as seen by the fact that it has the highest net heat release at 50% load, followed by B20–20% Pen RCCI and then Diesel, which has the lowest net heat release. At 75% load, the net heat emission that is produced by B20–20% Pen RCCI is the greatest, followed by that produced by DIESEL and B20–20% Pen RCCI. At particular loads, B20–30% Pen RCCI has the lowest net heat release, however at 100% load, B20–30% Pen RCCI has the biggest net heat release, followed by B20–20% Pen RCCI.

## Summary

This research study proposes a solution for CRDI engines to utilise a variety of waste cooking oils. Furthermore, in order to offer Reactivity Controlled Compression Ignition (RCCI),which guarantees that the diesel engine functions as efficiently as feasible. Waste cooking oil was successfully prepared in compliance with the standards for the manufacture of biodiesel.

Following the creation of the high performance mix, its physical and chemical properties were evaluated. The high performance fuel mixture is tested utilizing a range of charges under a variety of loads. The thermal efficiency of the brakes was observed to be fairly high for the B20–30% Pen RCCI. The volumetric efficiency of B20–10% Pen RCCI was found to be relatively good, but that of diesel was low. The B20–30% Pen RCCI has shown improved emission characteristics, including a decrease in carbon monoxide and carbon dioxide emissions over a wide range of load circumstances. Diesel has the lowest NOx emissions imaginable, followed by B20–30% Pen RCCI. The amount of hydrocarbon emission was highest in the case of RB20–30%.

## Data Availability

The authors state that all necessary data to replicate their findings can be found inside the content of the research.
